# Involvement of community health workers in the COVID-19 pandemic response in Uganda: A qualitative study

**DOI:** 10.1371/journal.pgph.0003312

**Published:** 2024-06-21

**Authors:** David Musoke, Micheal Jonga, Gloria Kisakye Ndagire, Benon Musasizi, Amanuel Gidebo, Asrat Tolossa, Maya Thomas, Peter Waiswa, Richard Rumsey

**Affiliations:** 1 Makerere University School of Public Health, Kampala, Uganda; 2 World Vision, Kampala, Uganda; 3 World Vision, Mississauga, Ontario, Canada; Brigham and Women's Hospital, UNITED STATES

## Abstract

Community Health Workers (CHWs) are a key human resource for health particularly in low- and middle-income countries. In many parts of the world, CHWs are known to have played an instrumental role in controlling the COVID-19 pandemic. This study explored the involvement of CHWs in the COVID-19 response in Uganda. A qualitative study that involved 10 focus group discussions (FGDs) among CHWs was conducted. The study was carried out in 5 districts of Amuria, Karenga, Kamwenge, Bugiri and Pader. The FGD guide used explored the role of CHWs in the COVID-19 response in their communities including lived experiences, challenges, and coping mechanisms. The data were analyzed thematically with the support of NVivo version 12 pro (QSR International). CHWs were at the frontline of COVID-19 prevention interventions at households and in the community. CHWs raised awareness on prevention measures including wearing face masks, hand hygiene, and social distancing. They identified suspected cases such as new members entering the community, as well as individuals returning from abroad with signs and symptoms of COVID-19. CHWs mobilized the community and increased awareness on COVID-19 vaccination which played an important role in reducing misinformation. They also supported home-based management of mild COVID-19 cases through isolation of patients; provided health and nutritional guidance among patients in their homes; and referred suspected cases to health facilities for testing and management. Both monetary and non-monetary incentives were provided to support CHWs in the COVID-19 response. However, the adequacy and timing of the incentives were inadequate. Routine services of CHWs such as health promotion and treatment of childhood illnesses were disrupted during the pandemic. CHWs played an instrumental role in response to the pandemic especially on surveillance, risk communication, and observance of preventing measures. Strategies to ensure that routine services of CHWs are not disrupted during pandemics are needed.

## Introduction

Community health workers (CHWs) have delivered health services for several years particularly in low- and middle-income countries (LMICs) [1]. Well facilitated, equipped and trained CHWs contribute to resilience of community health systems [2]. As community members themselves, CHWs provide a unique opportunity to facilitate an effective linkage to care [3]. The roles of CHWs in many contexts extend beyond health promotion to community management of mild cases for example as part of integrated community case management of childhood illnesses (iCCM) of pneumonia, diarrhoea, and malaria, providing oral and injectable contraceptives, treating malnutrition, and administering vaccines among children [2–5]. CHWs have been reported to treat nearly 50% of malaria cases in Liberia and Rwanda [6,7]. They also play a major role in prevention and control diseases through health promotion at household and community levels [8–10]. Well-trained and managed CHWs are essential to harnessing the role of communities in health systems, supporting primary care, and contributing to health outcomes [10,11]. CHWs’ involvement in service delivery has been credited for reductions in morbidity and mortality of under 5 children, as well as for HIV, TB and malaria [4,12,13].

Uganda’s health system is structured around the national minimum health care package that aims to ensure all villages in the country have the capacity to mobilize individuals and households for better health outcomes [14]. CHWs, locally referred to as village health teams, form the basic structure of the health system that acts as the first contact of communities to the health sector [15]. Since inception of the program in Uganda, many CHWs have been trained in various districts across the country [15]. Although CHWs are volunteers in many countries including Uganda, reforms were proposed by the World Health Organization (WHO) to professionalize the cadre and remunerate them accordingly [16,17] hence providing a framework to optimize community heath. However, these reforms have not been adapted fully in many countries particularly in sub-Saharan Africa including Uganda.

CHWs contributed to previous responses to Ebola and Zika outbreaks as community educators and supporting community surveillance capabilities [18]. Community level interventions for COVID-19 response including prevention, surveillance, vaccination, and home-based management of mild cases have been recommended as part of the national community engagement strategy in Uganda [19]. In implementing the strategy, CHWs were recommended to be at the forefront of the community response to the pandemic. It is believed that CHWs were instrumental in the COVID-19 response across the country. However, it is not clear to what extent CHWs participated in supporting these activities in the community to respond to COVID-19. In addition, the challenges known for many years to affect CHWs such as minimal training, lack of remuneration, poor supervision, and lack of supplies [20–22], could have affected their performance during the pandemic. There is hardly any evidence on the contribution of CHWs in the control of COVID-19 in the country. This study therefore explored the involvement of CHWs in the COVID-19 response in Uganda.

## Methodology

### Study design and participants

A qualitative study in September 2022 was conducted using focus group discussions (FGDs) among CHWs in Uganda. We conducted 2 FGDs in each of the 5 districts hence a total of 10 FGDs. These FGDs were sufficient to reach data saturation. Each FGD was comprised of an average of 10 participants that reflected on their involvement in the COVID-19 response. The 2 FGDs per district were conducted in various sub-counties that were randomly selected. Each village in Uganda is mandated to have 4 CHWs among whom 2 are particularly involved in iCCM (malaria, diarrhoea and pneumonia). CHWs participated in the study whether they were engaged in iCCM or not. These CHWs are volunteers hence only occasionally receive non-financial incentives such as t-shirts from the MOH and supporting partners.

### Study area and setting

This study was conducted in the World Vision Uganda (WVU) CHW program areas in the country. WVU has been supporting community health in 18 districts across the country (Bundibugyo, Kiboga, Kakumiro, Oyam, Kyankwanzi, Rakai, Buliisa, Tororo, Busia, Kamwenge, Amuria, Bugiri, Buikwe, Agago, Pader, Karenga, Mayuge and Terego). According to the Uganda Bureau of Statistics regional classification [23], these districts fall within 10 regions of Bukedi, Bunyoro, Buganda, Teso, Lango, Toro, Busoga, West Nile, Karamoja and Acholi. Initially, 50% of the 10 WVU regions were selected randomly hence involving 5 of them. From the 5 regions, 1 district was involved in the study (for regions with more than 1 district, randomly selection was done). Among the selected districts, 5 sub-counties were randomly selected to take part in the study. For this study, 5 randomly selected regions (Bukedi, Karamoja, Toro, Busoga and Acholi) were involved. The corresponding randomly selected districts involved in the study were Amuria, Karenga, Kamwenge, Bugiri, and Pader respectively. The five districts have varying land sizes, populations, number of households and main economic activities. Amuria, has a land size of 950 sq km, a population of 270,928, and main economic activities of agriculture and livestock. Karenga, has a land size of 7,223.70 sq km, a population of 51,533, and main economic activity of agriculture. Kamwenge, has a land size of 2,439.40 sqkm, a population of 270,668 and its main economic activities are agro-processing and commercial farming. Bugiri, has a land size of 1,046 sq km, a population of 382,913, and main economic activity of agriculture. Pader has a land size of 3,362.50 sq km, a population of 178,004 and its main economic activity is agriculture [24].

### Data collection

An FGD guide was developed and administered among CHWs to assess their involvement in the prevention and control of COVID-19 in their communities. The guide explored lived experiences, challenges, and coping mechanisms of CHWs while performing their roles during the pandemic such as surveillance, COVID-19 vaccination, home-based management of cases, and referral of patients. The FGDs were facilitated by a facilitator and a notetaker, and lasted 60 minutes on average. All FGDs were conducted in a central place in the area (such as residence of the local leader) in the local language of the locality by experienced and trained research assistants. These research assistants had bachelors degrees in health related disciplines and were trained for 2 days which included pretesting the tool. The rest of the research team, who had vast experience in qualitative research, were involved in this study. During data collection, the research assistants were closely supervised by 2 researchers (MJ–MPH and DM–PhD, both male). In addition, daily debriefing meetings were held to ensure data quality. COVID-19 standard operating procedures for small groups were observed including social distancing, hand washing, and use of face masks.

### Data management and analysis

All the FGDs were audio recorded during the sessions. These audio files were then transcribed verbatim in the local language by the research assistants and later translated to English. Once the transcripts were verified, analysis was done thematically using the inductive approach with the support of NVivo version 12 pro (QSR International). The thematic analysis involved the researchers reading the transcripts several times to familiarize themselves with the data. Thereafter, words or related phrases from the transcripts were grouped together to form codes. These codes were used to generate a code book that supported the analysis. The data was coded by two independent members of the research team (MJ and DM) who are experienced qualitative researchers. Related codes were then grouped to form sub-themes, and related sub-themes grouped to form themes. Selected quotes from the transcripts were obtained for use in the presentation of results.

### Ethical considerations

Approval to conduct the study was obtained from the Makerere University School of Public Health Research and Ethics Committee (SPH-2022-306) and the Uganda National Council for Science and Technology (HS2502ES). Administrative clearance was obtained from the respective districts before commencement of the study. All study participants provided written informed consent at the initiation of data collection.

## Results

Seven main themes emerged from the data: health promotion; surveillance; COVID-19 vaccination; home-based management of mild COVID-19 cases; referral of COVID-19 cases; motivation of CHWs; and CHW routine services during the pandemic.

### Health promotion

CHWs were involved in the promotion of various practices in preventing the spread of COVID-19 at household and community levels. These practices included awareness creation on COVID-19 clinical presentation, social distancing, self-isolation, hand washing with soap, and use of face masks. CHWs were also at the forefront of mobilizing community members for various interventions such as distribution of government face masks.

*“We gave out masks as well which were supplied by the government through the sub county*. *During the distribution of masks*, *we created awareness about COVID-19 including how to avoid getting the disease*.*”* participant, FGD 4

While implementing the COVID-19 preventive activities, CHWs used megaphones, flyers, community meetings, and other avenues. Promotion of hand washing by CHWs in some settings involved a house-to-house strategy to construct facilities such as “tippy taps” (locally made handwashing facilities). Demonstrations were also carried out by some CHWs for skills transfer for example during construction of hand washing facilities. Promotional materials on COVID-19 signs and symptoms used by CHWs were provided by either government agencies, implementing partners, or private donations from politicians and other well-wishers. In addition, CHWs were generally exemplary by wearing masks, carrying out social distancing, and observing good hygiene practices.

*“I made a recording that could teach people about COVID-19 so I walked with a loud speaker as the people were hearing about the virus and how to protect themselves*.*”* participant, FGD 2

There were various experiences on adherence to COVID-19 standard operating procedures as many youth did not believe that disease was real, and wearing of face masks was resisted in some instances. However, CHWs persisted with health education till most community members started to adopt these interventions. In some communities, these behavioral changes were undermined by several challenges including high cost of soap and lack of face masks. It was also hard to get hand sanitizers in the community so other alternatives such as local alcohol brew were adopted in some settings. There was a group of people who feared to wear masks particularly among those with respiratory conditions such as asthma.

*“At the time when the pandemic was at its peak*, *we mobilized our people to maintain social distancing and avoid being in large crowds*. *We also told them that if they felt any kind of illness*, *they needed to stay at home*. *We mobilized them to wash their hands and also encouraged them to put on masks*.*”* participant, FGD 6

CHWs got their information about COVID-19 from various sources including some who participated in training organized by implementing partners in the various districts, radio talk shows, and health workers from nearby facilities.

“*We were trained on COVID-19 at the health center IV which was very beneficial to us*. *Thereafter*, *we went and shared what we learnt with other people to enhance their practices on preventing COVID-19 in the community*.*”* participant, FGD 5

### Surveillance

CHWs were involved in active surveillance through identification of suspected cases and contact tracing. This activity involved investigating suspected individuals such as new visitors in their communities, those returning from abroad, or alerts of family members that were showing signs and symptoms of COVID-19. In case of any death in the community, this was treated as a suspected COVID-19 related fatality hence triggering immediate reporting for testing and contact tracing to the nearby health facilities. In the eventuality that a suspected case was confirmed to be COVID-19 positive, isolation was carried out in their homes for those with mild symptoms, and family members tested. These activities were greatly supported by CHWs in the community, working closely with health practitioners from facilities in the area.

*“We sensitized community members on the signs of COVID-19 so these people were very alert and in case they saw any one with flu and cough*, *they immediately reported to us*, *so that one of us would go and visit that person*.*”* participant, FGD 2

Telephones and temperature guns were commonly used in community surveillance of COVID-19 by CHWs. Indeed, telephone calls were the main channel used in communicating with higher administrative levels particularly health workers to report suspected COVID-19 cases, management of suspected cases, or requesting for logistical assistance for surveillance or referral.

*“Once a person was identified to have COVID-19*, *the health assistant would call and tell you that their family should be isolated*, *and no one should be close to them until the heath professional provided further guidance via the phone*.*”* participant, FGD 3

### COVID-19 vaccination

CHWs were primarily involved in mobilization of community members for COVID-19 vaccination, either through house-to-house visits or using megaphones. In addition, they sensitized community members about the benefits of vaccination and vaccine safety. Many CHWs had to get vaccinated to prove to the community that it was safe.

*“During the vaccination of COVID-19*, *we as CHWs acted as an example as we got vaccinated first then the rest of the people were keen to see what happened to us*. *But when they saw that we were not dying*, *they eventually also got vaccinated*.*”* participant, FGD 2

Misinformation and misconceptions surrounding the COVID-19 vaccine were reported by the CHWs which were mainly propagated through rumors. This misinformation included becoming infertile in both women and men, exacerbation of existing conditions, and death. The misinformation instilled fear in the minds of many people in the community. Other members of the community feared getting vaccinated due to side effects that their colleagues shared with them such as headache, body weakness, and vomiting. However, CHWs were instrumental in carrying out health education for example through health campaigns on radio and print media to mitigate the misinformation. Other factors that supported CHWs in enhancing COVID-19 vaccine acceptance were the requirement to be vaccinated to access public offices, as well as the need to reopen the economy and schools.

*“That negative information on COVID-19 vaccination was a lot including rumors that after one got vaccinated*, *they would spend a few days and then die which really scared the community*. *But one day*, *a CHW went and received the vaccine*, *and many people observed to see if anything happened to him*. *When nothing happened to him*, *many people decided to go for vaccination*.*”* participant, FGD 7

### Home-based management of mild COVID-19 cases

Home-based management of mild cases was not reported in all the districts as some communities did not get many cases of COVID-19. The home management strategies that the CHWs were involved in included isolation, as well as guidance and counselling of the infected people and their families. For the COVID-19 patients being managed at home, the health advice passed on by CHWs included exercising, staying positive about recovering from the disease, wearing face masks, and good nutrition.

*“Health workers taught and allowed us to monitor mild cases of COVID-19 at home mainly through enforcement of isolation of the infected person*. *I used to tell patients to do exercise around their homes and to wear a mask*. *I also used to do guidance and counselling to the patients*.*”* participant, FGD 7

However, some of the experiences around home management of mild cases were not good. Indeed, there were instances of discrimination against entire families, COVID-19 patients, and the CHWs that were offering support. It also emerged that certain households were stigmatized and denied access to social services such as water sources during home-based care of COVID-19 patients. This attitude was mainly driven by the fear to contract COVID-19.

“*I had a case on home-based management where a COVID-19 patient and his family were self-isolating in the community*. *The family was discriminated against even at the water sources*, *and whenever they needed water*, *they had to come to me for support*.*”* participant, FGD 2

Another experience shared by CHWs was that in some communities, it was difficult to keep the mild cases isolated as they would escape to the general population.

*“The most difficult time during COVID-19 was when we were faced with this one COVID-19 patient and after knowing his status*, *he decided never to stay home as he walked all over the village drinking and interacting with whoever he found as if he wanted to spread the virus to everyone*.*”* participant, FGD 4

### Referrals of COVID-19 cases

CHWs supported referral of COVID-19 cases to nearby health facilities after identifying the suspects. The district taskforces provided logistical support to transport suspected cases to the nearest health facility. Standby vehicles and ambulances were available in many of the districts. Telephone contacts of the district taskforces including the chairpersons were shared widely among the community including CHWs which also facilitated the referral process.

*“We referred COVID-19 patients by calling the health facility staff*. *The vehicles at the districts and health centres were usually available with fuel to support with the transportation of cases*.*”* participant, FGD 3

In places where an ambulance was not readily available, “boda bodas” (commercial motorcycles) were used to transport suspected cases to the health centre for assessment.

*“I know of a case where the family members had to sit on a boda boda to the hospital for further COVID-19 examination as there was no ambulance in our area*. *I also know two people who developed COVID-19 and had to be transported to the hospital on boda bodas*.*”* participant, FGD 4

### Motivation of CHWs

Monetary and non-monetary incentives were provided to motivate the CHWs during the pandemic. Non-monetary incentives including health educational and promotion materials, and kits were provided by the MOH to some CHWs, while others received megaphones. The CHW kit was comprehensive and contained several items such as a thermometer, pulsimeter, masks, aprons, gumboots, torch, t-shirt, and reading mat. However, it was noted that the adequacy and timing of these incentives were insufficient. In addition to MOH, implementing partners also supported COVID-19 interventions to motivate CHWs. The support was offered in the form of bags, thermometers, village health registers, reporting forms, gumboots, umbrellas, first aid kits, and allowances. Uniforms for CHW identification were provided by a few partners in some of the study districts.

“*We had some logistical support from the Ministry of Health and other partners especially in observing the standard operating procedures*. *For example*, *some non-governmental organizations gave us soap*, *masks*, *disinfectants*, *and sanitizers”*. Participant, FGD 10

### CHW routine services during the pandemic

CHW routine services were disrupted by the COVID-19 pandemic. These services were affected as the tasks for CHWs were shifted to focus on the COVID-19 response. Routine services in maternal health, iCCM, and hygiene promotion were among those disrupted during the pandemic. It emerged that CHWs suspended general health promotion activities such as household sanitation improvement, and promotion of antenatal care during the COVID-19 response.

*“Before the pandemic*, *we used to encourage people to maintain hygiene in the community*. *We used to tell them to dig latrines*, *have rubbish pits*, *and encourage them to take their children for immunization but all these were not happening because of the COVID-19 pandemic*.” participant, FGD 5

## Discussion

CHWs played an integral role in response to the COVID-19 pandemic in Uganda. They provided preventive, curative and rehabilitative services to their communities amidst poorly resourced circumstances. Incentives (monetary or non-monetary) and logistics were either inadequate or not provided in a timely manner. As grassroots actors, CHWs leveraged their unique position in the communities and social networks to translate directives into contextually appropriate messages and actions. These actions ensured adherence to COVID-19 standard operating procedures such as social distancing, hand washing, wearing face masks, and mobilization for vaccination. The COVID-19 pandemic showed that strong community structures are crucial for timely pandemic response [2]. Community engagement in pandemic response requires adequate funding as well as being organized. Health systems can only institute and maximize preventive strategies when implemented through well trained, resourced, and prepared CHWs. Future pandemic preparedness and response efforts ought to prioritize building on successes of resilient community interventions, with CHWs considered a key human resource in these settings [25].

CHWs form the basic structure of health systems that act as the first contact of communities to services [26,27]. Findings from our study indicate that CHWs were at the frontline of prevention interventions at households and in communities during the pandemic. Their roles included awareness creation on COVID-19, enforcement of standard operating procedures (social distancing, hand washing, and wearing face masks), community sensitization on vaccine safety, and mobilization for COVID-19 vaccination. The awareness creation channels used by CHWs included house-to-house campaigns, print media, demonstrations, and being exemplary. Participation of CHWs in outbreaks in various parts of the world is not new [5,28,29]. A review of response to the Ebola 2014 and Zika 2015 epidemics showed that CHWs played critical roles prior to and during the occurrences [18]. CHWs have particularly been involved in increasing access to health services, communicating health concepts in a culturally appropriate fashion as community level educators, and contributing to surveillance systems [18,30]. In resource constrained countries such as Uganda, CHW involvement should be the foundation for holistic community-based interventions while responding to pandemics.

CHWs in our study were involved in active community surveillance of COVID-19 particularly identification of suspected cases and contact tracing. Contact tracing is a particularly key activity of any pandemic response as demonstrated in several studies [31,32]. This surveillance, which entailed strengthening the link between communities and higher levels of the health system, was vital in responding to the pandemic. Evidence from Thailand shows that CHW participation in community surveillance enabled a robust response to the COVID-19 pandemic without the use of a costly country-wide lockdown or widespread testing [33]. Community based surveillance systems are as important as national centralized systems while responding to outbreaks. Having appropriate tools such as for communication including telephones was key during community surveillance. This communication was seen between CHWs and community members, as well as between CHWs and health practitioners. Due to the importance of community structures, efforts to build a resilient health system requires significant investment in strengthening community-based surveillance systems to promote early detection and prevention of future pandemics [25].

CHWs were also involved in mobilizing the community for COVID-19 vaccination, as well as increasing health awareness on the benefits of the intervention. These findings are similar to existing literature that CHWs play a major role in prevention and control of diseases through community mobilization at household and community levels [8,9]. This role of creating awareness and mobilization was important to reduce misinformation about COVID-19 vaccination in the community. Vaccine hesitancy and misinformation on COVID-19 standout as a recurring issue that was of global concern during the pandemic [34,35]. Indeed, several studies have documented misinformation during the COVID-19 pandemic [36,37]. For example, a study carried out in northern Uganda articulates how rumors spread very fast, and CHWs were mobilized to conduct sensitization [38]. Poor access to information, culture and religion, and poor health literacy have been shown to contribute to misinformation during pandemics [37]. A study on COVID-19 messages being forwarded on *WhatsApp* (a social media platform) in Singapore found that 35% of the messages were based on falsehoods, and 20% mixed truth and false claims [39]. Misinformation has a great impact on health outcomes and behavior of people during pandemics. A randomized controlled trial also found that exposure to online misinformation on COVID-19 vaccine reduced vaccination intention in the UK and USA [40]. Misinformation is therefore a serious threat to public health especially during outbreaks. It is therefore important to create health literacy and improve access to culturally appropriate messages during health promotion campaigns of pandemic response. CHWs, if well trained, can spearhead communicating accurate and clear information to communities during outbreaks.

Motivation of CHWs is critical in sustaining community health interventions. However, evidence shows that CHWs often lack support, and their retention seems to persistently remain challenging. CHWs need additional logistical and motivational support to consistently deliver on their roles [4] particularly during outbreaks. In our study, we found that both monetary and non-monetary incentives were provided to support CHWs during their response to the pandemic. However, the adequacy and timing of the incentives seemed to be of particular concern. It is known that non-monetary motivation can improve performance of CHWs. Indeed, studies carried out in Uganda and Mozambique found that non-cash interventions (training, supervision and logistical support) were effective in enhancing CHW performance in Wakiso district [9,41]. Evidence during the COVID-19 pandemic also shows that proper motivation of CHWs enhanced their contribution to the response in various parts of the world [30,42]. Therefore, timely and adequate support to CHWs is needed to ensure their full potential during response to pandemics including COVID-19 is realized. In the long term, having CHWs fully institutionalized and receiving adequate remuneration in Uganda as recommended by the WHO would enhance their performance including during pandemics.

Participation of CHWs in the COVID-19 response significantly disrupted the routine roles and responsibilities of CHWs such as treatment of childhood illnesses (malaria, diarrhoea, and pneumonia) and hygiene promotion. Neglecting such major public health interventions during a pandemic could have both short and long term implications [43]. Whereas short term effects could include increased morbidity and mortality, long term consequences of disruption of CHW services such are poor growth and development among children may be realized. Evidence from the West African Ebola outbreak showed that disruptions in measles vaccination resulted in increased incidence that persisted after the outbreak [43,44]. In addition, there has been an increase in zero dose children during the COVID-19 pandemic particularly in LMICs [45]. Pandemic response needs to recognize the importance of continuity with routine services in the health sector particularly at community level. Strategies to ensure continuity of such services among CHWs during pandemics are therefore important. These strategies could include providing logistical and supervisory support to CHWs related to their routine roles when responding to pandemics.

The study had some limitations such as being conducted in districts that were supported by World Vision Uganda hence the findings may not be generalizable to the entire country. In addition, demographic information of the participants was omitted during data collection. However, the study was carried out in various regions of the country which provided a diverse range of insights which could inform future research on pandemic management as well as policy, practice, and programming in Uganda.

## Conclusion

CHWs played an instrumental role in response to the pandemic especially on surveillance, risk communication, and observance of preventive measures. However, the incentives received for their involvement in the pandemic were inadequate. CHWs need more support towards their involvement in response to pandemics including COVID-19. Strategies to ensure that CHW routine services are not disrupted during pandemics are also needed.

## Supporting information

S1 Checklist COREQ checklist. (DOC)

S1 Table Data output. (DOCX)
