# Supplementary material for: Involvement of community health workers in the COVID-19 pandemic response in Uganda: A qualitative study
Source: PLOS Glob Public Health. 2024 Jun 21;4(6):e0003312. doi: 10.1371/journal.pgph.0003312 (PMC11192370; doi:10.1371/journal.pgph.0003312)
Supplement: S1 Table — (DOCX) [file pgph.0003312.s002.docx]

**Roles and responsibilities of community health workers during COVID-19 pandemic response**

| **Role** | **Description** |
| --- | --- |
| **Health promotion and communication** | - Strategic communication, creating awareness, information and education to gain and hold trust of the communities. - Thus promotion of various practices in preventing the spread of COVID-19 at household and community levels. - These practices included awareness creation on COVID-19 clinical presentation, social distancing, self-isolation, hand washing and mobilization for COVID-19 mass vaccination - VHTs were instrumental in carrying out health education to mitigate the misinformation. |
| **Surveillance** | - CHWs were involved in community based surveillance and case detection including deaths. - CHWs were involved in active surveillance through identification of suspected cases and contact tracing. - This activity involved investigating suspected cases such as new visitors in the community, those returning from abroad, or alerts of family members that were showing signs and symptoms of COVID-19 |
| **Home based case management** | - Supporting self-isolation, community-based drug distribution and referrals as appropriate. - The home management strategies that the VHTs were involved in included isolation, as well as guidance and counseling of the infected people and their families. - For the COVID-19 patients being managed at home, the health advice passed on by VHTs included exercising, staying positive about recovering from the disease, wearing a face mask, and good nutrition. |
| **Referrals** | - CHWs supported referral of COVID-19 cases to nearby health facilities after identifying the suspects. - The district taskforces provided logistical support to transport suspected cases to the nearest health facility. - Standby vehicles and ambulances were available in many of the districts. Telephone contacts of the district taskforces including the chairpersons were shared widely among the community including CHWs which also facilitated the referral process. |
| **CHW routine services during the pandemic** | - CHW routine services were disrupted by the COVID-19 pandemic. - These services were affected as the tasks for CHWs were shifted to focus on the COVID-19 response. - Routine services in maternal health, iCCM, and hygiene promotion were among those disrupted during the pandemic. - It emerged that CHWs suspended general health promotion activities such as household sanitation improvement, and promotion of antenatal care during the COVID-19 response. |
